# Emboli

**Published:** 1871-05

**Authors:** Walter Hay

**Affiliations:** 169 Dearborn street; Chicago


ARTICLE V.—*Emboli.* By WALTER HAY, M.D., Chicago.

Mrs. P——, (104 Forquer street,) aged about 35 years; multipara; about two years since had a miscarriage accompanied by severe hæmorrhage, about six or eight weeks subsequent to which, she came under my professional observation. Her appearance and history indicated the existence of perfect health prior to the accident just mentioned. Her condition, when first seen was characterized by anæmia, great prostration of strength, dyspnea, (increased in the horizontal position) coldness of extremities, increased vaso-motor energy (indicated by diminution in the calibre of the arteries and hardness and sharpness of the pulse), diminution of the respiratory murmur, and the presence of dry râles throughout the lungs, increased irritability and debility of the heart, with bruit de soufflet at the base, bowels acting nearly normally, slightly constipated, urine loaded with amorphous urates or soda and ammonia, with some purpurine (no albumen), frequent, short dry cough.

We have here, in the presence of the abundance of urates and purpurine in the renal secretion, and in the diminished temperature, the evidences of deficient oxydation, as a proximate cause of which, we recognize the decreased blood-supply to organs resulting from diminished caliber of the arteries, and the pulmonary engorgement, lessening the respiratory surface and impeding the free interchange of gases in the lungs. For the next step in the chain of causes, we must look to the disturbance in the co-ordination between respiration and circulation; the blood was returned to the lungs by the right side of the heart, in excess of the capacity of the left side for its removal and transmission through the systemic circulation; this increased carbonization of the blood moreover perpetuating itself by its reaction upon the nervous system. We are thus led to the last in the series of pathological lesions, the primary cause of the whole,—regurgitation from deficiency of the aortic valves.

The therapeutic indications suggested obviously by the above analysis, were to increase oxydation and hence excretion,—which necessarily involved increase in the blood-supply to excretory organs, and this could be effected only by adjusting the disturbed equilibrium between cardiac and arterial action, *i. e.*, by diminishing arterial and increasing cardiac energy.

To this end, the following formulæ were prescribed and administered:

℞. Tinct. Opii, - - - } āā gtts. xxx.

Tinct. Digital. Pur., - - }

Every four hours, and

℞. Potass. Iodid, - - - - grs. viii.
Syr. Scillæ, - - - - - ʒj. M.

Every alternate four hours.

At the end of eight days, treatment was discontinued, the patient resuming her usual avocations.

Since the time above referred to, this patient has had five or six seizures, almost identical to that already described, in which the same plan of treatment yielded similar results.

Six months ago, exposure to wet induced inflammatory action in the pleura of the left side, followed by considerable effusion, which was arrested and removed by Hydrarg. Chlor. Mit. gr. ss., Pulv. Scillæ Mar., grs. ij, every four hours; and a blister to diseased side, followed during the subsidence of the inflammation and the process of absorption, by Lugol's Solution applied externally, and in five drop doses every six hours, internally.

March 13, 1871. Found patient in a similar condition to that described in the beginning of this report, and into which she had already several times relapsed, which condition yielded to the treatment already described in the same connection by the 16th, when she was again discharged.

March 27, 11 A. M. Patient had passed sleepless night; the countenance was anxious, the extremities cold, the pulse small and hard and hurried, the breathing difficult, there was frequent, short dry cough, the tongue was moist and coated with dead epithelium, the bowels were constipated, there was great nausea, the urine, when cold, depositing urates copiously, the respiratory murmur was replaced by dry râles, the heart's action was tumultuous, resembling the irregular roll of drum, the relations of sounds could not be distinguished; the patient complained that her heart was "*squeezed.*"

Diagnosing endocarditis, directed Hydrarg. Chlor. Mit. gr. ss.,

Pulv. Scillæ Mar. grs. ij., every four hours, and the precordial region to be painted with Lugol's Solution.

March 28, 11 a. m. Bowels had been evacuated five or six times, urine more copious, paler and clearer, the urates disappearing under the increased supply of oxygen; pulse larger, softer, slower; breathing more free, some moist râles perceptible; "*squeezing of the heart better.*" Treatment of yesterday to be continued, with six hours interval between doses. Ten drops of Lugol's Solution every alternate six hours.

March 29th. Symptoms of yesterday all favorably modified; continue treatment.

March 30th. Slept well last night, feels much better; symptoms all improved; turns upon either side easily. Omit pills, continue Lugol's Solution. Feels some soreness about right shoulder extending from middle of clavicle to middle of deltoid muscle, which she attributes to pressure, as she lay upon that side last night.

March 31st. Right arm swollen to an enormous size from shoulder to ends of metacarpal bones; mottled with purple blotches, cold, pulseless at wrist and axilla; great tenderness from middle of clavicle to insertion of the deltoid, the region of greatest intensity being lower border of axilla.

But one cause could be assigned for this condition, the arrest of the circulation in the subclavian by an embolon. And this diagnosis was confirmed by Dr. Morris J. Asch, U. S. A., who was kind enough to examine the case with me. Upon this hypothesis, the therapeutic indications appeared to be, to sustain cardiac and to diminish arterial energy—that the impacted mass might thus be driven farther away from the center of circulation, and the area deprived of its blood-supply be diminished, in the hope of gaining time for the establishment of collateral circulation; to this end, Tinct. Opii, Tinct. Digitalis, pur. āā, ʒ ss., and Lugol's Solution, gtts. x, were given every alternate four hours; the arm being kept wet with a solution containing Plumb. Acetat, grs. x; Tinct. Opii, ʒ j, to the ounce of water, and enveloped in cotton wool.

The condition of the patient gradually improved under this treatment until April 4, when the circulation of the arm was fully re-established, and the limb diminished to nearly its normal size.

April 5, 11 A.M. Pain and tenderness in the middle of the left forearm; hand and wrist slightly swollen and cold; pulse, at axilla, feeble, at bend of elbow and wrist entirely absent; dry râles in lungs. The treatment of the 31st ult., etc., which had been partially discontinued, was resumed, with addition of Syr. Scillæ, ʒj, to each dose of the opium and digitalis.

April 7, 10.30 A.M. Circulation in both arms normal; respiration freer than at any previous period in the history of the case; general condition much improved, no bad symptom remaining except tumultuous action of the heart. Left patient at 10.52 A.M. in excellent spirits, laughing; at 11 (within eight minutes) she was dead. There was not the slightest struggle or motion. Was the aorta obstructed by a clot? No autopsy permitted.

169 Dearborn street.

